# Mechanical Reinforcement of Polyamide 6 by Cold Hydrostatic Extrusion

**DOI:** 10.3390/ma14206045

**Published:** 2021-10-13

**Authors:** Monika Skorupska, Mariusz Kulczyk, Sylwia Przybysz, Jacek Skiba, Jan Mizeracki, Joanna Ryszkowska

**Affiliations:** 1 Institute of High Pressure Physics, Polish Academy of Sciences (Unipress), Sokołowska 29/37, 01-142 Warsaw, Poland; mariusz@unipress.waw.pl (M.K.); sylwia@unipress.waw.pl (S.P.); skiba@unipress.waw.pl (J.S.); janekm@unipress.waw.pl (J.M.); 2 Faculty of Materials Science and Engineering, Warsaw University of Technology, ul. Wołoska 141, 02-507 Warsaw, Poland; joanna.ryszkowska@pw.edu.pl

**Keywords:** cold hydrostatic extrusion, polyamide 6, mechanical properties, structure

## Abstract

This paper presents the effect of severe plastic deformation obtained using the cold hydrostatic extrusion (HE) method on the mechanical and structural properties of polyamide 6 (PA6). As a result of the plastic strain, a significant increase in ultimate tensile strength and tensile modulus were observed. Tensile strength rose by almost 500%, up to the level of 508 MPa, whereas the tensile modulus rose by about 65%. Flexural modulus increase was also observed to 3230 MPa, i.e., by approx. 160%. As a result of high plastic deformation, the structure of the polyamide 6 changed significantly, as evidenced by its fibrous nature as presented in the results of the scanning electron microscopy inspection (SEM). The surface quality of products investigated was tested using profilometry.

## 1. Introduction

Hydrostatic extrusion (HE) is one of the techniques used to obtain severe plastic deformation (SPD) in order to change a material’s structure, as well as to alter mechanical and physical properties of metals and metal alloys. The effect of severe plastic deformation in metals is the transformation of a coarse-grained structure into an ultra-fine-grained or nanometric structure [1,2,3,4,5,6]. Many studies have shown that the refinement of the structure in HE processes leads to a significant improvement in the strength of metals and their alloys, such as aluminium, copper and their alloys [1,2], iron [3], titanium [4], steels [5] or cast iron [6]. Due to the deformation of the material in the HE processes using high hydrostatic pressure, the generation and propagation of cracks is inhibited, so it is possible to maintain the materials’ cohesion, even at a very high level of strain, and thus drastically increase their strength and plasticity.

The HE processing of polymers was first described in 1964 by Pugh and Low [7]. Inoue et al. [8] described the HE processes for, inter alia, high-density polyethylene (HDPE), poly(vinyl chloride) (PVC), acrylonitrile-butadiene-styrene copolymer (ABS), polypropylene (PP), polyamide 6 (PA6), polyoxymethylene (POM) and poly(methyl methacrylate) (PMMA). HE processes were carried out with reduction in (R) within the range of 1–10 (R is the ratio of the cross-section area before and after the HE processes) at a temperature of 50–90 °C. The dependence of the extrusion pressure (p_HE_) on the R ratio was analysed, confirming their commonly known linear dependence [9]. The linear dependence of p on R was also confirmed, inter alia, for polyethylene (PE) and PP in the works of Buckley and Long [10]; Davis [11] for PE; and Yoon et al. [12] and Ariyama et al. [13] for PP. They found that for amorphous polymers: poly(methyl methacrylate) (PMMA) and high impact polystyrene (HIPS), HE has a significant impact on their thermal properties. The strength tests of HDPE, PP, PVC and ABS subjected to the HE process were described by Inoue et al. [14]. The polymers were subjected to tensile tests to 90% at room temperature, obtaining a PP strength of 50 MPa.

Coates et al. [15] presented the mechanics of the HE in the solid phase through a conical die of polyethylene (HDPE) at 100 °C and POM at 164 °C. They have shown how the knowledge of the tensile stress–strain–strain rate dependence for each polymer can be used to explain the observed experimental behaviour of these materials in the process of solid-phase extrusion. In the paper by Hope et al. [16], the behaviour of two grades (homopolymer and copolymer) of PMMA during HE at 90 °C was reported. It was concluded that in the case of crystalline polymers, it proved necessary to take into account the effects of strain hardening, strain rate and pressure dependence of the flow stress when analysing the mechanics of the extrusion process.

Strength tests of amorphous polymers after HE processes in the temperature range of up to 100 °C for R reduced to 3 are described in Inoue et al. [17], where for polycarbonate (PC) they obtained a yield strength (σ_y_)—35 MPa. Inoue and Nishihara [18] described the HE parameters and their influence on the mechanical and physical properties of polymers, including PE, PP, PA6, ABS, PVC, polystyrene (PS).

Ladizesky et al. [19] investigated the process of cold hydrostatic extrusion of the PE composite with hydroxyapatite with the reduction to R = 11 using rods obtained by mixing the powders and pressing them on a screw press at a temperature between 210 °C and 250 °C. For pure PE, after extrusion with the reduction R = 4, the maximum flexural modulus (E_f_) was 2500 MPa, and the flexural strength (σ_fM_) 52 MPa, while for PE filled with 30% by volume of hydroxyapatite, the corresponding properties were 5200 and 63 MPa, respectively, which clearly indicates the strengthening nature of the filler used. In the publication of Kozlov et al. [20] a conventional extrusion of charges made of PE powder was carried out at temperatures above 120 °C, where the influence of PE anisotropy on hardness after deformation was demonstrated. In the study by Jin et al. [21] a biodegradable poly(L-lactide) (PLLA) polymer after HE processing at 145 °C with reductions to R = 12 was described. The maximum σ_fM_ was 350 MPa and E_f_ = 7500 MPa, which is attributed to the strong orientation of the crystalline PLLA fibres caused by the HE processing.

PA6 polyamide is used in various industries such as transport, electronics, construction and packaging. It can also be a good alternative to metal when filled with glass fibre [22]. It can also be used in medicine, including in limb prosthesis processes for various types of prostheses: cosmetic [23] and functional [24,25,26], including kinetic, myoelectric and bionic prostheses. The currently produced functional prostheses are always heavier than cosmetic prostheses, which require a disabled person to change the habits acquired during the use of cosmetic prostheses. This fact is a significant problem in the case of prostheses for children, as movement symmetry disturbance may contribute to spinal problems. Tubes and rods acting as tibia and fibula bones are components of the leg prosthesis structure. These components are used as load-bearing structural components when walking or running. Metal materials are used for production of these components, such as stainless steel, aluminium and titanium steels [27], polymer thermoplastics, e.g., PP [28,29,30], but also carbon fibre reinforced polymer matrix (CFRP) composite [31]. However, other groups of materials are being sought that allow for the introduction of new, cheaper solutions for the construction of prostheses to make them more accessible to users, especially for children as they require frequent replacement.

Polyamide 6 is a material approved by the American Food and Drug Administration (FDA) and is widely used as a biomaterial in humans [32,33,34]. For example, in the study by Cossard et al. [35], the possibility of using PA6 with glass fibre as external bone fracture stabilisers known as “Illizarov rings” was investigated.

As part of the study, the suitability of HE for the modification of PA6 rods was investigated, in order to obtain components with increased strength properties, without modification of their chemical composition. However, to the best of the present study’s authors’ knowledge, no information has been ever published on PA6 cold-deformed in the solid state using the HE method in a form that would allow its commercial use, e.g., in the form of a rod. The aim of this study was to investigate the influence of variable parameters of the HE processes on the structure and properties of PA6.

## 2. Materials and Methods

### 2.1. Material

The initial state material used was unmodified extruded polyamide PA6 in the form of a rod with a diameter of 15.6 mm, purchased from MEGA-TECH s.c., Grodzisk Mazowiecki, Poland [36]. The properties of the PA6 polyamide before HE processing are given in Table 1.

### 2.2. Hydrostatic Extrusion

**Figure 1:**
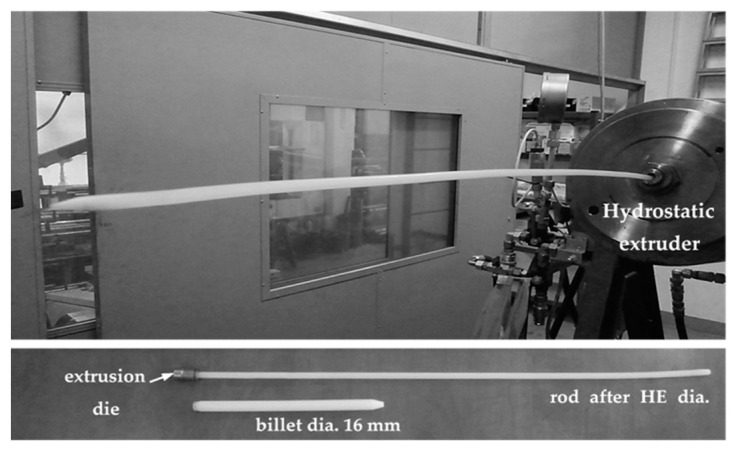
Polyamide 6 (PA6) after hydrostatic extrusion (HE): HE presses and example dimensions of the initial charge and the rod after HE.

The principle of the HE method is presented in the work by Pachla et al. [37]. Rods with a diameter of 15.6 mm made of PA6 polymer were subjected to the process of cold hydrostatic extrusion using a press designed and manufactured at the Institute of High Pressure Physics of the Polish Academy of Sciences (Warsaw, Poland). The press, with a working chamber of 22 mm, enables extrusion at pressures of up to 2 GPa. Prior to the HE processes, the rods were coated with a silicone oil-based grease (Dow Corning Corporation, Midland, MI, USA) and squeezed out at a linear speed (v) in the range of 3.5–111 mm s^−1^ through forming a matrix with die the vertex angle 2α = 45° and diameters of 6, 7 and 8 mm (Figure 1).

In order to avoid the effects of strong heating of the material during plastic deformation, the plastic strain zone and the material extracted from the die were intensively cooled with cold water. It was observed that the diameter of each rod after the hydrostatic extrusion process was larger than the diameter of the die. This is a phenomenon similar to the Barus effect, so-called post-extrusion swelling [38]. Depending on the die used, a variable degree of reduction ratio (R) and true strain (ε) are obtained. The reductions (R), calculated as the ratio of the diameter before and after the HE processes, correspond to the true strains (ε) in the range of 0.68–1.90, where the true strain is given by the natural logarithm of the extrusion ratio R. During the HE processes, a strong adiabatic heating effect occurs (AH), which is the result of changing the mechanical work of pressing into heat. The adiabatic temperature (T_ad_) used to describe the parameters of the HE process was estimated using the following Formula (1) [39]:

(1) $$T_{\mathrm{ad}}=\beta \frac{W}{c\rho }=\beta \frac{p}{c\rho }$$

where: W—mechanical work of plastic deformation performed during extrusion (J); p_HE_—extrusion pressure (MPa); *c*—specific heat (J/g K) and ρ—density (g cm^−3^) and β—share of mechanical work that has been converted into thermal energy (0.95 was adopted for the HE processes).

In the case of polyamide PA6 deformed at room temperature within an extrusion pressure range between 80 and 430 MPa, ρ = 1.14 g cm^−3^ and *c* = 1.7 J/g K, the adiabatic temperature increase Δ*T* fell within the range of 50 to 211 °C.

The PA6 polymer is a hygroscopic material and has a tendency to absorb water. Due to the necessity of using water cooling in the HE processes to limit the effect of released heat on the properties of the final product, the materials were cured for 14 days after completion of the HE processes.

### 2.3. Experimental

The roughness tests were carried out using the HOMMEL TESTER T8000 profilometer by Hommelwerke (Villingen-Schwenningen, Germany) and a HOMMEL-ETAMIC TKU300/600 contact head with an apex radius of 2 μm. The head travel speed was 0.5 mm/s. The roughness parameter $R_{a}$ was obtained, i.e., the arithmetic mean value of the ordinates Z(x) of the roughness inside the elementary Section l. This value was calculated by the TurboWave software (V7.4, 2015, Hommelwerke GmbH, Villingen-Schwenningen, Germany) using Equation (2):

(2) $$R_{a}=\frac{1}{l}\int \;\left|Z\left(x\right)\right|dx$$

As part of the research, a series of five measurements was performed using the contact method. The measuring sections were 4.8 mm long. Roughness profiles were obtained for each of the measurements.

The static tensile test (Zwick/Roell Z250, Ulm, Germany) was performed for samples with a diameter of 5 mm and a length of 10 mm at a tensile speed of 1 mm·min^−1^ to determine the tensile strength σ_M_ in MPa and elongation at break ε_M_ in %. A 1-axis compression test (Zwick/Roell Z250, Ulm, Germany) using samples with the diameter of the extruded rod and the height equal to 1.5 times the diameter was performed with a compression speed of 1 mm min^−1^. The compression test was continued until the sample was compressed by 70% of its original height in order to determine the compressive modulus E_c_ in GPa, compressive stress at deformation 0.1% σ_0.1_ in MPa, and compressive strength σ_M_ in MPa. A 3-point bending test (Zwick/Roell Z250, Ulm, Germany) was performed using samples with a diameter of 5 mm and a length of 160 mm, with a spacing between supports of 125 mm, a maximum deflection of 69 mm and a bending speed of 2 mm min^−1^ in order to determine the flexural modulus E_f_ in MPa, flexural strength σ_fM_ in MPa, and the flexural strain at maximum flexural strain ε_fM_ in mm. An impact tensile toughness test was carried out using an Instron Dynatup 9250 HV testing machine (Norwood, MA, USA) on samples with a diameter of 5 mm and a length of 10 mm with an impact energy of 50 J and an impact speed of 1.8 ms^−1^ in order to determine the Charpy impact toughness K in kJ m^2^. At least 3 samples were used for each mechanical test. The test results of the PA6 rods subjected to the cold hydrostatic extrusion process were compared with the properties of the initial state rods (non-deformed) with a diameter of 15.6 mm.

Structural analysis was conducted using a Scanning Electron Microscopy—ZEISS SEM microscope, model ULTRA PLUS, with a Gemini column (Jena, Germany). The fracture surfaces were analysed on samples frozen for 5 min in liquid nitrogen and broken through bending. Before the observations, the samples were dusted with a layer of gold.

The DSC analysis of PA6 was performed using a differential scanning calorimeter DSC Q1000 (TA Instruments, New Castle, DE, USA). Samples (ok. 5 mg) were closed in hermetic aluminium cups and heated at 10 °C/min in the temperature range 0 to 250 °C.

Moisture content w was determined by Equation (3):

(3) $$w=\frac{m-m_{1}}{m_{2}}$$

where: m and $m_{1}$ are the mass of the weighing bottle with the material sample before and after drying, respectively, and $m_{2}$ is the mass of the investigated material sample.

The density (ρ) test was conducted using hydrostatic weighing, on samples of the same dimensions, in accordance with ISO 2781.

## 3. Results and Discussion

### 3.1. Hydrostatic Extrusion of Polyamide PA6 with Quality of Surface Analysing

**Figure 2:**
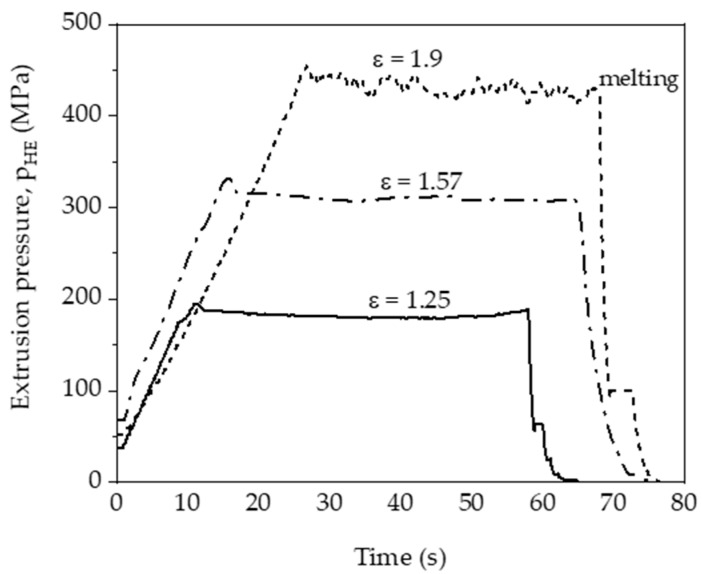
Pressure characteristics of the PA6 polymer cold hydrostatic extrusion process for selected degrees of plastic deformation ε.

During the HE processes, the change in process pressure as a function of time was registered. Example pressure characteristics of the hydrostatic extrusion process for three degrees of plastic deformation ε are summarised in Figure 2.

At the beginning of the extrusion process, pressure jumps (‘breakthrough pressure’) are observed (Figure 2), which are the result of changing the static (higher) friction to kinetic (lower) friction. The increase in p_HE_ caused by the increase in ε is a well-known phenomenon characteristic of metals and metal alloys, polymers, composites, etc. The increase in p_HE_ during deformation with ε > 1.25 (Figure 2) is caused by an increase in the packing density of the polymer macromolecules. The pressure then stabilises. The flat runs of the pressure change curve during the process indicate a constant, linear rate of extrusion of the product, which is a necessary condition for obtaining a homogeneous structure along the entire length of the rod and the resulting repetitive physical and mechanical properties [40]. The constant rate of extrusion determined on the basis of the flat courses of the pressure change curves was recorded for samples tested at a true strain of 1.25 and 1.57.

**Figure 3:**
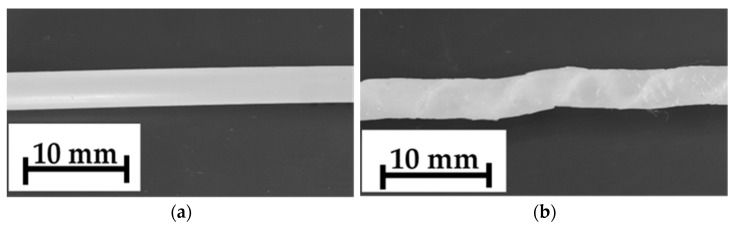
Image of the rod structure subjected to the cold HE process: (**a**) D_1.57_5 (ɛ = 1.57) and (**b**) E_1.90_7 (ɛ = 1.9).

However, at a true strain ε = 1.9, the pressure was not stable and the product after the HE process was inconsistent (Figure 3).

**Figure 4:**
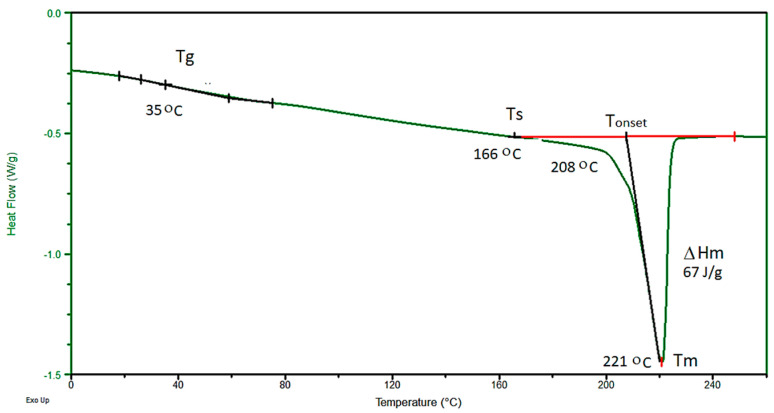
DSC thermal curve of the PA6 sample A. Where: differential scanning calorimetry (DSC), glass transition temperature (T_g_), temperature where melting process begins (T_s_), initial melting temperature (T_onset_), melting point temperature (T_m_), melting enthalpy (ΔH_m_).

Lack of pressure stability during the HE process for sample E_1.90_7 is caused by high p_HE_, causing the T_ad_ temperature to rise to 211 °C. In order to explain the reasons for the occurrence of defects in the profile of sample E_1.90_7, a DSC analysis of sample A—PA6 was performed before the HE process (Figure 4).

**Figure 5:**
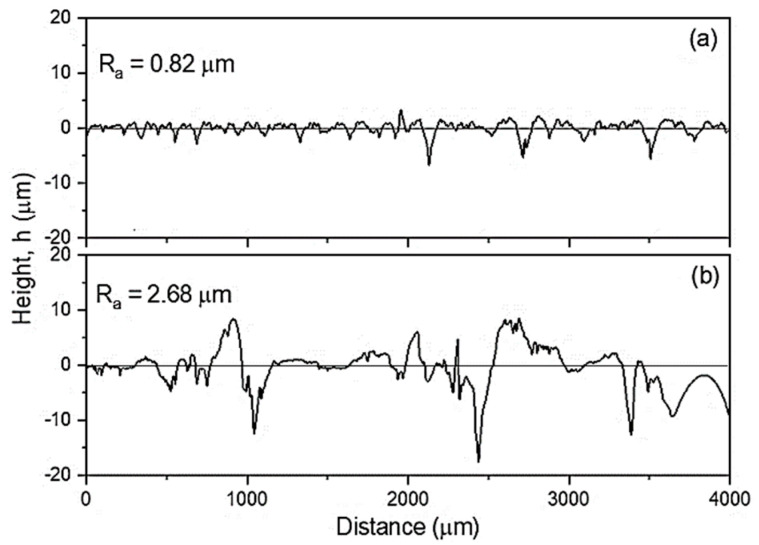
Comparison between surface roughness dependence *R_a_* on cold hydrostatic extrusion parameters of polyamide PA6, sample: D_1.57_5 (**a**) and E_1.90_7 (**b**).

Based on the course of the (DSC) curve, the glass transition temperature (T_g_) was determined, which was 35 °C. The analysed HE processes of the PA6 polymer were performed at adiabatic temperature in the range of T_ad_ = 40–211 °C, so at a temperature above the T_g_ level. The second transformation in the DSC thermal curve is the endothermic transformation of the polymer crystalline phase melting. This transformation is described by the initial melting temperature (T_onset_ = 208 °C), the melting point temperature T_m_ = 221 °C and the melting enthalpy ΔH_m_ = 67 J/g. The melting process begins at around 166 °C (T_s_). Since the T_ad_ of the sample E_1.90_7 subjected to the HE process is higher than the melting process beginning, some of the crystallites melt during the HE process. The consequence of this is the formation of a porous and rough surface on the rod after the HE process. When the HE process pressure is stable, the rods are characterised by a smooth surface with low roughness along the entire length (R_and_ = 0.82 μm) as is shown in Figure 5a for the sample D_1.57_5. For the sample E_1.90_7 (Figure 5b) with ε = 1.9, when the pressure during the HE process was unstable, the surface of this sample had a much greater roughness (R_and_ = 2.68 μm), and spiral cracks were noticed on the extruded rod.

**Figure 6:**
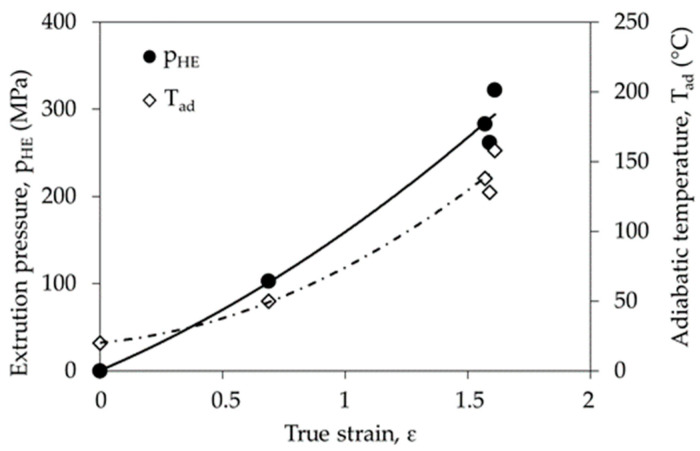
Dependence of pressure (p_HE_) on true strain (ε) for the cold HE processed PA6 polymer at different levels of true strain.

**Figure 7:**
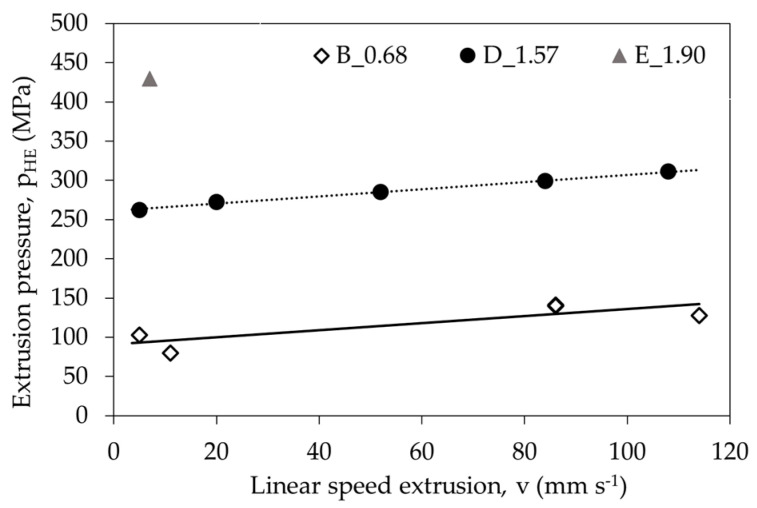
Dependence of pressure (p_HE_) on the linear extrusion rate (v) for the cold HE processed PA6 polymer at different levels of true strain.

For the same values of plastic deformation with increasing extrusion rate (v) an increased pressure during the HE process was observed (Figure 6). The effect of the pressure increases when the HE processes speed is increased is similar, regardless of the strain value. The pressure change as a function of the strain was also analysed for the same cold HE process speed v = 5 mm s^−1^ (Figure 7). It was found that increasing the degree of plastic strain results in the occurrence of a significantly higher pressure during the plastic deformation process.

Plastic deformation of the PA6 polymer with true strain ε ~ 0.7 increases the process pressure to approx. 100 MPa, which is accompanied by an increase in the adiabatic temperature T_ad_ to about 70 °C., so at the true strain value ε ~ 1.6 the pressure reaches the value of approx. 320 MPa, and the T_ad_ of the material is 153 °C. To achieve such a high T_ad_ temperature, it is necessary to supply much more heat than in the case of ε ~ 0.7. The level of heat supplied depends on the specific heat of the material. The specific heat is the heat needed to change body temperature by one unit per unit mass of the material:

(4) $$c=\frac{\Delta Q}{m\;\Delta T}$$

where: c is the specific heat (J/kg K), ΔQ—heat delivered (J), m—material mass (kg), and ΔT temperature difference (K).

The specific heat of the PA6 polymer amounts to 1700 J/kg K and is over 4 times higher than the specific heat of Cu (385 J/kg K). This means that in order to increase its adiabatic temperature during the HE processes, it is necessary to provide much more thermal energy for the same volume of PA6 polymer than in the case of Cu. To generate such a large amount of thermal energy in the material for the extrusion process, a much greater extrusion pressure must be used. Moreover, the PA6 polymer has a low thermal conductivity λ = 0.24–0.28 W/m K, i.e., the ability to conduct heat, so under the same conditions much less heat is removed from it than, for example, from Cu (400 W/m K), which is a material of over 1500 times greater thermal conductivity. Therefore, the PA6 polymer cools down slowly, holding its temperature much longer, which causes the heat to act on the material for a longer time, promoting changes in its structure and, consequently, its properties.

### 3.2. Mechanical Properties

**Figure 8:**
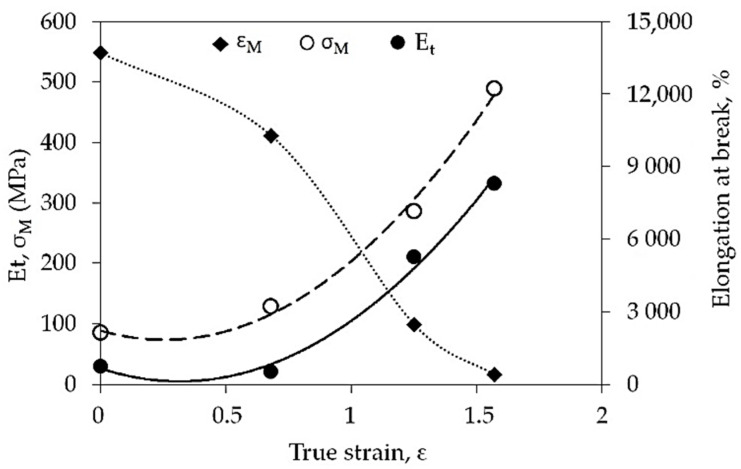
Relationship of mechanical properties determined the tensile test as a function of strain after PA6 cold hydrostatic extrusion (HE). Where: tensile modulus (E_t_), elongation at break (ε_M_), tensile strength (σ_M_).

**Figure 9:**
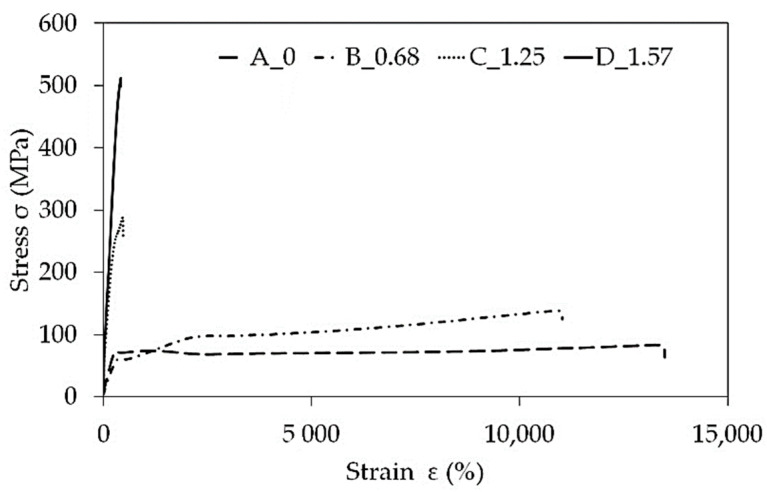
Statistic uni-axial tensile characteristics for PA6 before hydrostatic extrusion (A) and after (B, C, D).

Changes in the mechanical properties of the PA6 polymer after the hydrostatic extrusion process, and measured statistic tensile tests, are shown in Figure 8 and Figure 9. 

The percentage changes in the mechanical parameters are also summarised in Table 2.

In the analysed cold HE processes, it was observed that the tensile modulus (E_t_) and tensile strength (σ_M_) of materials increases with the degree of true strain. At maximum true strain value ε = 1.57 an over 10-fold increase in the tensile modulus and an almost 5-fold increase in tensile strength was observed (Table 2). As the degree of strain degree increases, the value of strain at break decreases to the level of ε_M_ ~ 380% for the true strain ε = 1.57. Figure 9 shows a significant increase in the strength of PA6 with the degree of true strain during the HE process, while decreasing elongation. The higher increase in strength of PA6 polymer indicates that the higher true strain during HE cold deformation is a key factor to promote the increase in crystallinity degree and homogeneity of the oriented structure, which will be discussed in the next article.

**Figure 10:**
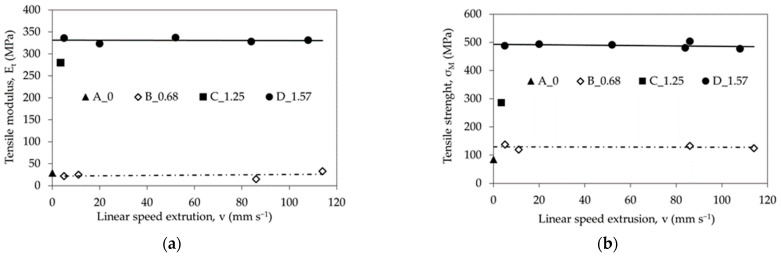
The influence of HE processes linear speed extrusion on mechanical changes: (**a**) tensile modulus (E_t_), (**b**) tensile strength (σ_M_).

It was also found that the tensile modulus, tensile strength and strain at break at the same strain value do not depend on the HE process linear speed extrusion, see Figure 10a,b.

Analysis of the mechanical properties determined in the compression test, bending test and impact strength test of the PA6 polymer as a function of the degrees of actual strain applied are given in Table 3.

With an increase in the degree of true strain during the HE processes, an increase in all measured mechanical properties is observed. However, compared to the initial state material (A), the values obtained in the compression test, i.e., the compressive modulus (E_c_) and the compressive strength (σ_M_), are lower over the entire range of strain values. However, the improvement in the mechanical properties is visible in the bending and impact tests. The increase in these features is not as significant as in the case of the features determined in the tensile test.

### 3.3. Comparison of Selected Mechanical Properties of PA6 Processed Using Various Methods

**Figure 11:**
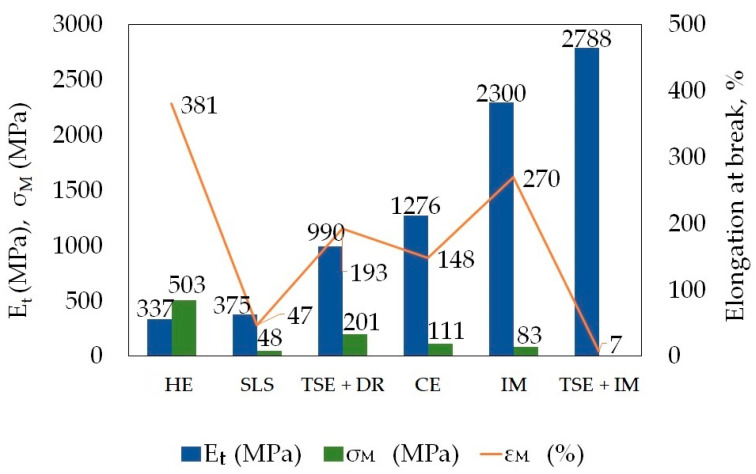
Comparison of the mechanical properties determined in the PA6 polymer tensile test after processing using different techniques. Where: hydrostatic extrusion (HE), selective laser sintering (SLS), twin-screw extrusion (TSE) and cold-drawing (DR), solid-phase extrusion (CE), injection moulding (IM).

Polyamide 6 can be processed with various techniques, including: selective laser sintering (SLS) [41], twin-screw extrusion (TSE) and cold-drawing (DR)—TSE DR [42], Equal-Channel Multiangular Extrusion (ECAP) [43], solid-phase extrusion (CE) [44], injection moulding (IM) [45,46] and twin-screw extruder + injection (TSE + IM) [47,48]. Figure 10 and Figure 11 show a comparison of selected properties of PA6 samples formed using different processing methods.

The tensile modulus of the PA6 samples after the HE process is similar to the modulus of the polymer processed using the SLS technique and is almost seven times lower than the modulus of samples processed with the IM technique (Figure 11), whereas the tensile strength is the highest in samples subjected to the HE process—six times higher than in samples processed with the IM technique. Samples subjected to the HE process have the highest elongation at break compared to the samples subjected to other processes to which they were compared.

**Figure 12:**
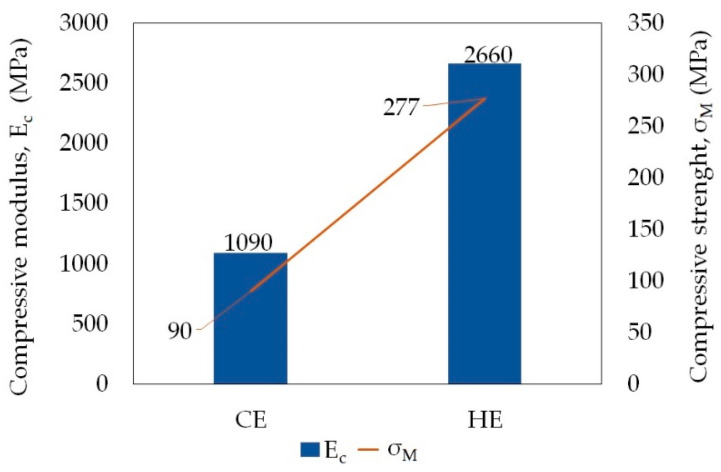
Comparison of the strength properties determined in the compression test of PA6 samples formed in the solid-phase extrusion (CE) and hydrostatic extrusion (HE) processes.

For most of the compared processing methods it was necessary to use a temperature above the PA6 melting point, which is >220 °C (IM, TSE, TSE + DR, TSE + IM, or slightly lower, i.e., ~195 °C (SLS, CE), or 150 °C (ECAP). During the HE processes, the PA6 material remained solid and was not additionally heated. The changes that took place in the material were caused by the heat generated by high pressure, i.e., the mechanical work of extrusion converted into heat. The compressive modulus of the PA6 samples subjected to the HE process is more than two times greater, whereas the compressive strength is more than three times greater than in the polymer samples subjected to the CE process [49]. As with the properties in the static tensile test, these changes are the result of changes in the structure of the materials (Figure 12).

### 3.4. Structural Investigations

**Figure 13:**
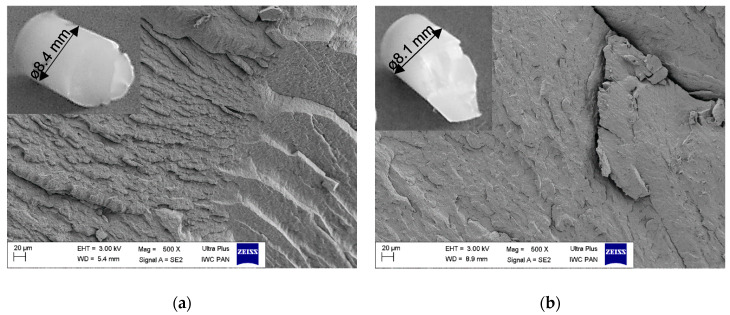

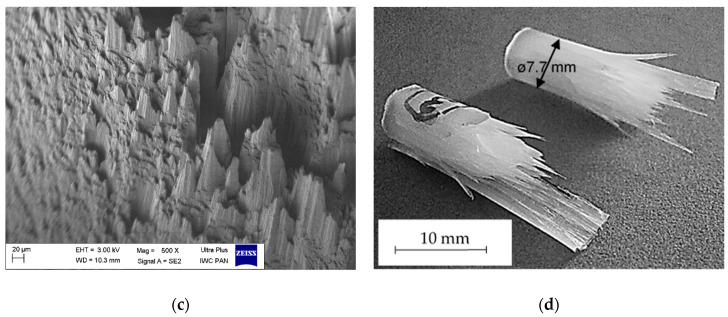
Scanning electron microscopy (SEM) images of cryo-fractured transverse cross-sections of the polyamide PA6 in (**a**) initial state, (**b**) rod after cold hydrostatic extrusion HE with true strain ε = 0.68 and linear extrusion speed v = 5 mm s^−1^, (**c**) rod after cold HE with ε = 1.57 and v = 5 mm s^−1^, and (**d**) macro image of (**c**). Note: insets in (**a**,**b**) shows the macro images of (**a**,**b**).

Figure 13 shows polyamide PA6 cryo-fractured in liquid nitrogen (LN2) transverse cross-sections of the initial state, Sample A (a), the polyamide PA6 subjected to the HE process with the same v ~ 5 mm s^−1^ and two different degrees of strain: ε = 0.68, Sample B_0.68_5 (b), and over 2 times greater ε = 1.57, Sample D_1.57_5 (c and d). Samples were cooled in liquid nitrogen (Messer Polska Sp. z o.o., Warsaw, Poland) and broken. Fractures of significantly different structure were observed. The initial state material samples (Figure 13a) and samples of material subjected to the HE process with ε = 0.68 (Figure 13b) have characteristic brittle fractures, typical of samples with a high degree of crystallinity and low impact strength [50]. The fracture of the samples made of initial state material (sample A) during the TSE process structure of the PA6 is oriented. The consequence of such formation of the structure in samples A is the formation of the stepped structure of the fracture. In the samples of the material subjected to HE process with a true strain ε = 0.68, the structure undergoes a distinctive change compared to the material subjected to the TSE process. The structural elements of this sample are finer and more regularly distributed, whereas samples of the material subjected to HE process with a true strain ε = 1.57 show a fibrous material structure (Figure 13c).

Fibres arranged in layers in the direction of extrusion are shown in Figure 13c,d. During the hydrostatic extrusion process, the PA6 macromolecules are drawn in the extruding direction. The distribution of fibres within the entire PA6 polyamide rod diameter is even, but a solid layer formed by fibres was found around the rod circumference (Figure 13d). This may be due to the shear forces occurring during the HE processes, which increase as the die angle increases. The shear force was greater at the circumference zone of the rod, which, together with the strongest adiabatic heating effect (AH) in the die/polymer subsurface layer, led to a strong coalescence of molecular chains and the structure presented (Figure 13d). The layer on the circumference of the PA6 polyamide rod becomes stiffer than the rod core and hence the rod breaks in a characteristic manner (Figure 13d).

The initial analyses of PA6’s structural changes before and after the HE process are described in Supplementary Materials. Based on these studies, it was found that the reason for the changes in strength properties is the increase in the degree of crystallinity and the increase in physical cross-linking with hydrogen bonds connecting PA6 chains after hydrostatic extrusion. (Figures S1 and S2, Tables S1 and S2 from Supplementary Material).

## 4. Conclusions

As part of the study, the influence of variable parameters of the hydrostatic extrusion process on the mechanical properties of PA6 was analysed. The rods subjected to the HE process were strained with variable true strain values in the range of 0.68 to 1.9. The extrusion process was accompanied by adiabatic heating effects increasing the temperature during the process to values exceeding the melting point at the true strain ε = 1.9. The process performed under these conditions does not allow a regular-shaped bar with low roughness to be obtained, as part of the PA6 crystalline phase melts during its course. Based on the analyses conducted, it can be concluded that the solid-phase cold hydrostatic extrusion process should be carried out under conditions generating T_ad_ higher than the T_g_ of the PA6 material and preferably below the temperature at which the crystalline phase begins to melt (T_s_). Such conditions occurred in the strain range of 0.68 to 1.57.

As the true strain value increases, material with a greater tensile modulus, tensile strength, flexural modulus and flexural strength is obtained. It also has a lower deformation at break. PA6 subjected to the HE process with a true strain ε = 1.57, compared to the material in its initial state, i.e., after the twin-screw extrusion process Sample A, it has a tensible modulus that is ten times higher, tensile strength that is almost five times higher, and elongation at break that is 35 times lower. In the case of the properties determined during the compression and bending tests, the changes are not as significant. The modulus marked during the bending test increases by 28%. A decrease in compressive strength is, however, observed.

As a result of the analyses conducted, it was found that for a given strain, the process pressure increases with the increase in the linear extrusion rate, whereas the mechanical properties in the entire range of the strain tested as a function of the strain rate do not change significantly.

The changes in the properties of the PA6 polymer subjected to the HE process are the result of structural changes. At a true strain ε = 0.68, a more regular structure is formed in the material than in the samples made of the initial state material (A). In contrast, a higher true strain ε = 1.57 in the HE process causes the structural elements to be formed into fibres that are connected and arranged along the extrusion direction. Macroscopic images of broken rods show that their cross-section structure is heterogeneous.

In further work on PA6 subjected to solid state HE processes, analyses of the structure of materials using various techniques will be performed to explain the mechanism of its changes during this process. In addition, the heterogeneity of the structure on the cross-section of the extruded rods will be evaluated.

## Figures and Tables

**Table 1:** Initial mechanical properties of the PA6 polyamide.

Property	Value	Units
Tensile modulus, E_t_	28.9	MPa
Tensile strength, σ_M_	85	MPa
Compressive modulus, E_c_	2770	MPa
Compressive strength, σ_M_	388	MPa
Compressive strength at yield stress, σ_y_	80.8	MPa
Flexural modulus, E_f_	1960	MPa
Flexural strength, σ_fM_	61	MPa
Elongation at flexural strength, ε_fM_	7.2	%
Impact strength, K	478	kJ m^−2^

**Table 2:** Summary of the tensile test results and the strength properties analysis of PA6 after the HE processes with a different degree of true strain.

Series	True Strain, ε	Et, MPa	ΔEt, %	σ_M_, MPa	Δσ_M_, %	ε_M_, %	Δε_M_, %
A	0	29 ± 3	-	85 ± 5	-	13,715 ± 645	-
B	0.68	23 ± 5	0	129 ± 19	52	10,280 ± 240	−25
C	1.25	210 ± 35	620	286 ± 45	236	2460 ± 245	−82
D	1.57	332 ± 10	1045	489 ± 10	475	381 ± 35	−95

**Table 3:** List of strength properties determined in the compression test, bending test and impact strength test for individual PA6 test series subjected to the HE processes.

Series	True Strain, ε	E_c_, MPa	σ_M_, 70% Strain, MPa	E_f_, MPa	σ_fM_, MPa	K kJ/m^2^
A	0	2770 ± 300	388 ± 29	1960 ± 125	61 ± 4	478 ± 9
B	0.68	2560 ± 100	253 ± 19	2160 ± 150	63 ± 5	448 ± 35
C	1.25	2680 ± 200	201 ± 3	2390 ± 175	66 ± 3	463 ± 20
D	1.57	3300 ± 100	213 ± 13	3230 ± 73	69 ± 4	482 ± 8

Where: compressive modulus (E_c_), compressive strength (σ_M_), flexural modulus (E_f_), flexular stress strength (σ_fM_), impact strength (K).

## Data Availability

The data presented in this study are available on request from the corresponding author.

## Supplementary Materials

The following are available online at https://www.mdpi.com/article/10.3390/ma14206045/s1, Figure S1: DSC thermograms of PA6 samples before the HE process (PA6_A) and after the HE process (PA6_D), Figure S2: Results of the DMA analysis of samples PA6_A and PA6_D, Figure S3. Results of dynamic viscosity of PA6 samples using a rheometer; Table S1: Results of DSC thermograms analysis of tested samples, Table S2: DMA analysis results.
