# Supplementary material for: Mechanical Reinforcement of Polyamide 6 by Cold Hydrostatic Extrusion
Source: Materials (Basel). 2021 Oct 13;14(20):6045. doi: 10.3390/ma14206045 (PMC8537147; doi:10.3390/ma14206045)
Supplement: Supplementary file 1 [file materials-14-06045-s001.zip › materials-1377275-supplementary.pdf]

Supplementary Material

Mechanical Reinforcement of Polyamide 6 by Cold Hydrostatic Extrusion

Monika Skorupska [1,*], Mariusz Kulczyk [1], Sylwia Przybysz [1], Jacek Skiba [1], Jan Mizeracki [1] and Joanna Ryszkowska [2]

[1] Institute of High Pressure Physics, Polish Academy of Sciences (Unipress), Sokołowska 29/37, 01-142 Warsaw, Poland; mariusz@unipress.waw.pl (M.K.); sylwia@unipress.waw.pl (S.P.); skiba@unipress.waw.pl (J.S.); janekm@unipress.waw.pl (J.M)
[2] Faculty of Materials Science and Engineering, Warsaw University of Technology, ul. Wołoska 141, 02-507 Warsaw, Poland; joanna.ryszkowska@pw.edu.pl
* Correspondence: monikaw@unipress.waw.pl

Research on the Structure of Polyamide PA6 Before and After Hydrostatic Extrusion

Materials and Methods

The samples prepared from rods made of PA6 were tested before hydrostatic extrusion (HE) (PA6_A), and after the HE process, the sample from the D series (PA6_D). Samples for tests using a rheometer in the form of discs with a bar cross-section and a thickness of about 1.5 mm, were cut from the rods across the direction of their extrusion. Samples for thermal analysis using differential scanning calorimeters (DSC) were taken from the disk's centre. Thermomechanical test specimens, Dynamic Mechanical Analysis (DMA) with dimensions 60 mm × 8 mm × 1 mm were cut from the rod along the extrusion direction.

Research on the structure of PA6 before and after HE process was carried out using indirect methods (DSC and DMA analysis).

The DSC analysis of PA6 was performed using a differential scanning calorimeter DSC Q1000 (TA Instruments, New Castle, DE, USA). The measurements were carried out in a helium atmosphere in hermetic aluminium crucibles. Approx. 5 mg specimens were heated at the rate of 10 °C/min in a temperature range from −80 to 260 °C.

The thermo-mechanical properties of samples with dimensions 60 mm × 8 mm × 1 mm were analysed with a DMA Q 800 (TA Instruments, New Castle, DE, USA). A 3-point bending test was performed at 1 Hz frequency and 20 mm amplitude. The preload force was set at 1 N. The measurements were carried out in a nitrium atmosphere. Specimens were cooled at −70 °C for 5 min and heated at the rate of 3 °C/min in a temperature range from −70 to 200 °C.

The rheological properties of PA6 were assessed using an Ares Rotary Rheometer (TA Instruments, New Castle, DE, USA), using the geometry of two parallel plates with a diameter of 25 mm and a gap between them of 1 mm. The measurements were carried out in a convection oven (TA Instruments, New Castle, DE, USA) at a temperature of 235 °C.

Results

The thermal analysis results using DSC are shown in Figure S1, and the results of the analysis of these thermograms are summarized in Table S1.

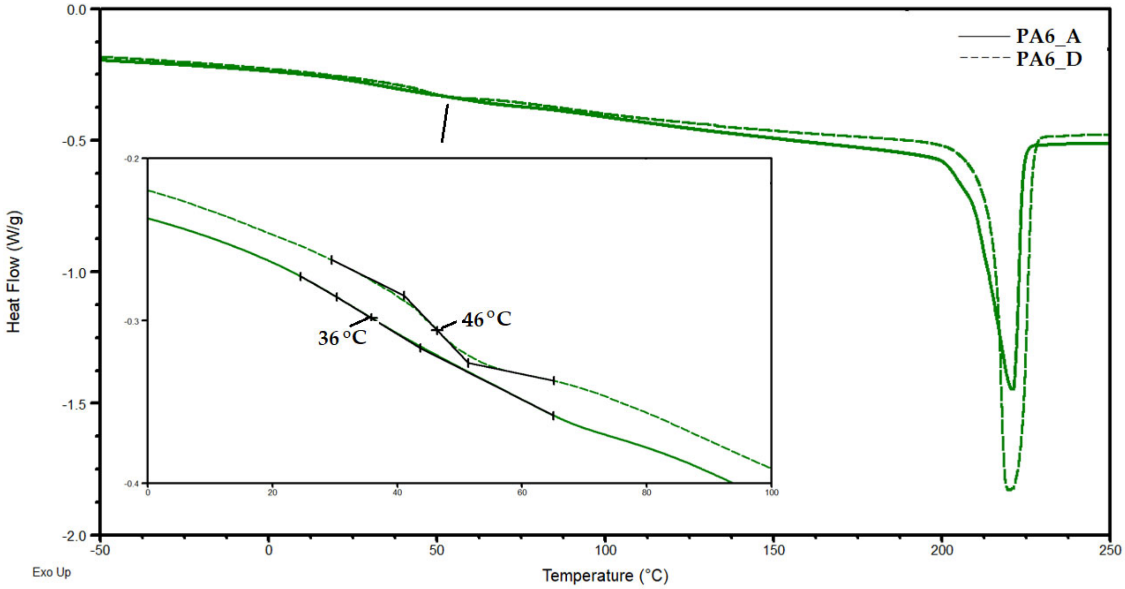

Figure S1. DSC thermograms of PA6 samples before the HE process (PA6_A) and after the HE process (PA6_D). Where: differential scanning calorimetry (DSC), Polyamide 6 (PA6), hydrostatic extrusion (HE).

Table S1. Results of DSC thermograms analysis of tested samples.

Sample	T_g, °C	T_m, °C	ΔH_m, J/g	X_c, %
PA_A	36	221	68.9	36.3
PA_D	46	220	86.7	45.6

Where: differential scanning calorimetry (DSC), glass transition temperature (T_g), melting point temperature (T_m), melting enthalpy (ΔH_m), crystallinity degree (X_c).

The degree of crystallinity was calculated based on the heat of melting for 100% crystalline polyamide 6 (ΔH_{m100} = 190 J g^{-1}) [1].

Based on the DSC analysis, it was found that the glass transition temperature (T_g) of PA6 before the HE process is approx. 36 °C and after the HE process, it is approx. 46 °C. The increase in the glass transition temperature results from a reduction in the mobility of the PA6 chains.

After the HE process, the sample's melting point (T_m) is slightly lower than that of PA6 in its original state. The samples differ in the enthalpy of melting (ΔH_m); the sample after HE is much higher. The polymer contains about 36% of the crystalline phase in the initial state and 46% after the HE process. As the T_g determined for PA6_A may raise doubts, the analysis using DMA was performed. The results of the DMA analysis are shown in Figure S2 and Table S2.

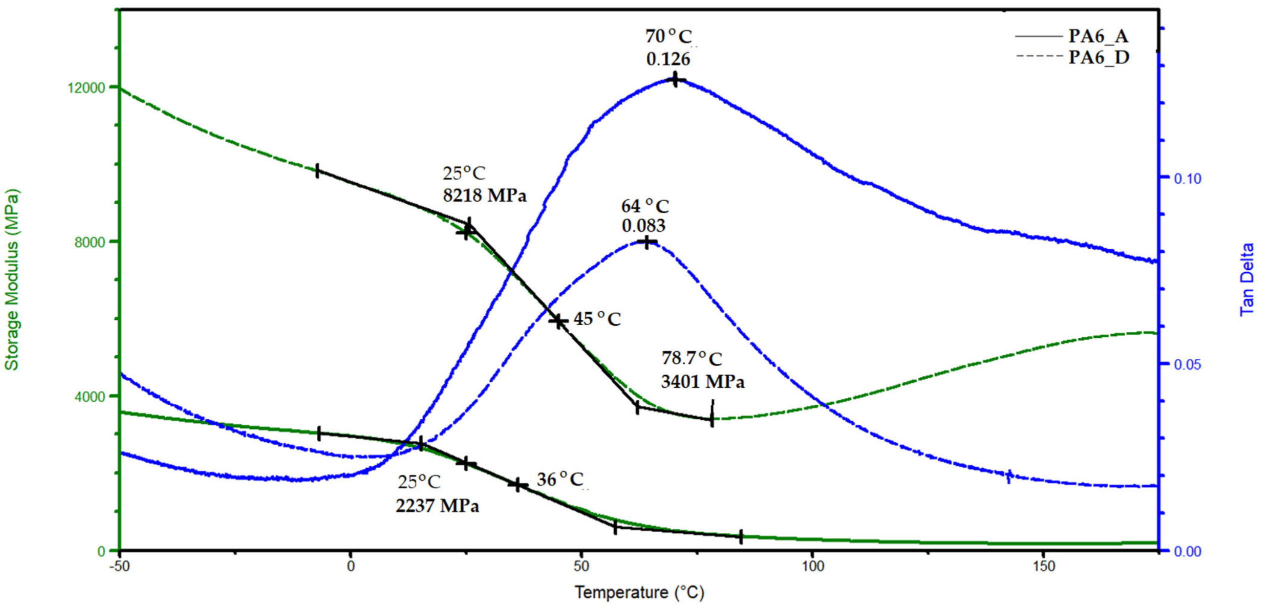

Figure S2. Results of the Dynamic Mechanical Analysis (DMA) analysis of samples PA6_A and PA6_D.

Table S2. DMA analysis results.

Sample	T_gE', °C	T at Tan Delta, °C	Tan Delta Max	E′ at 25 °C
PA_A	36	70	0.126	2237
PA_D	45	64	0.083	8218

Where: Dynamic Mechanical Analysis (DMA), storage modulus (E'), glass transition temperature (T_gE').

Based on the curves of changes of the storage modulus (E'), the T_g of both samples was determined. It is respectively 36 °C and 45 °C for PA6_A and PA6_D samples. It is similar to those determined based on DSC thermograms. The course of the E' curve indicates that cross-linking took place in the PA6_D sample, and it may be cross-linking with hydrogen bonds. The characteristic inflexion on the E' curve was observed at the temperature of 78.7 °C with a modulus of elasticity of 3400 MPa. The increase in cross-linking density resulting in a reduction in the mobility of macromolecules causes an increase in the T_g of PA6_D samples compared to the samples in the original state. The HE process reduces the vibration damping ability of the PA6_D sample.

Changes in the PA6 structure after the HE process were indirectly assessed by analyzing the rheological properties of the samples using the Ares rheometer. The rheological properties of polymers depend on molecular weight and chain structures: branching and crosslinking [2–4]. Figure S3 shows the complex viscosities of PA6_A and PA6_D.

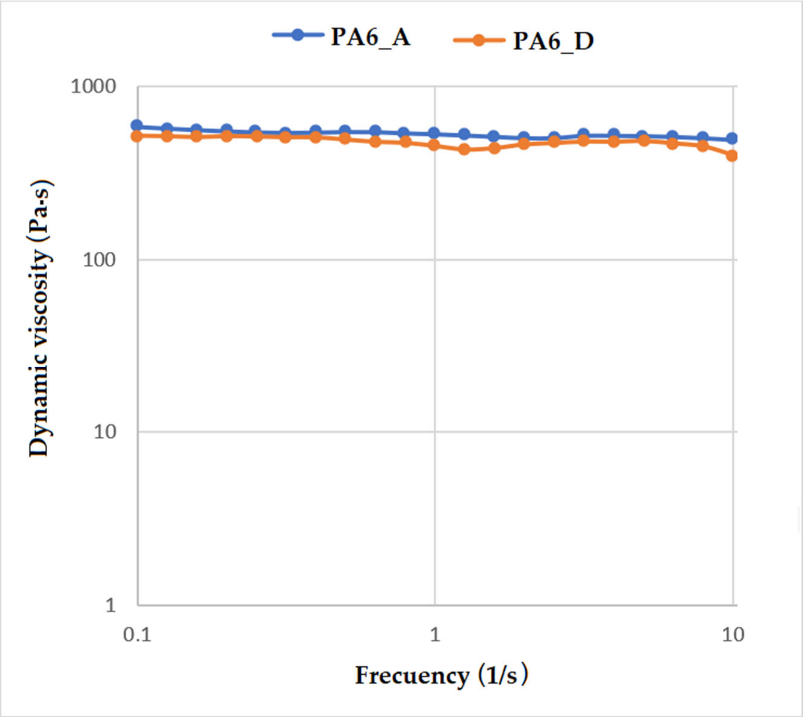

Figure S3. Results of dynamic viscosity of PA6 samples using a rheometer.

Dynamic viscosity of PA6_A changes with the change of the deformation frequency in the range of 585–490 Pa·s, and for the PA6_D samples in the range of 518–400 Pa·s, which is approx. 100 Pa·s less. This result indicates a significant reduction in the molecular weight of the PA6_D sample during the HE process.

Conclusions

During the hydrostatic extrusion process, the molecular weight of PA6 and the physical cross-linking of its macromolecules are reduced. These changes lower dynamic viscosity PA6, increase its T_g and the content of the crystalline phase. The consequence of these changes is a reduction in the ability to damp vibrations and an increase in the storage modulus of elasticity by approx. 270%.

References

1. Kaisersberger, E.; Knappe, S.; Möhler, H.; Rahner, D. *TA for Polymer Engineering: DSC, TG, DMA, TMA. Netzsch Annual for Science and Industry*, NETZSCH-Gerätebau GmbH: Selb, Germany 1994, v 3
2. Harrell, E.R.; Nakajima, N. Modified Cole–Cole plot based on viscoelastic properties for characterizing molecular architecture of elastomers. *J. Appl. Polym. Sci.* **1984**, *29*, 995–1010.
3. Kim, E.S.; Kim, B.C.; Kim, S.H. Structural effect of linear and star-shaped poly(lactic acid) on physical properties. *J. Polym. Sci Part B Polym. Physics*. **2004**, *42*, 939–946.
4. Scaffaro, R.; La Mantia, F.P.; Botta, L.; Morreale, N.; Dintcheva, N.T.; Mariani, P. Competition between chain scission and branching formation in the processing of high density polyethylene: Effect of processing parameters and of stabilizers. *Polym. Eng. Sci.* **2009**, *49*, 1316–1325.
